# Potential for diagnosis of infectious disease from the 100,000 Genomes Project Metagenomic Dataset: Recommendations for reporting results

**DOI:** 10.12688/wellcomeopenres.15499.1

**Published:** 2019-10-14

**Authors:** Gkikas Magiorkinis, Philippa C. Matthews, Susan E. Wallace, Katie Jeffery, Kevin Dunbar, Richard Tedder, Jean L. Mbisa, Bernadette Hannigan, Effy Vayena, Peter Simmonds, Daniel S. Brewer, Abraham Gihawi, Ghanasyam Rallapalli, Lea Lahnstein, Tom Fowler, Christine Patch, Fiona Maleady-Crowe, Anneke Lucassen, Colin Cooper

**Affiliations:** 1 Hygiene, Epidemiology and Medical Statistics, Medical School, National and Kapodistrian University of Athens, Athens, 11527, Greece; 2 University of Oxford, Oxford, UK; 3 Oxford University Hospitals NHS Foundation Trust, Oxford, UK; 4 Oxford Biomedical Research Centre, John Radcliffe Hospital, Oxford, UK; 5 University of Leicester, Leicester, UK; 6 Public Health England, London, UK; 7 Imperial College London, London, UK; 8 University of Cambridge, Cambridge, UK; 9 Swiss Federal Institute of Technology (ETH), Zurich, Switzerland; 10 University of East Anglia, Norwich, UK; 11 Earlham Institute, Norwich, UK; 12 Genomics England, London, UK; 13 Faculty of Medicine, University of Southampton, Southampton, UK

**Keywords:** metagenomics, full-genome sequencing, pathogens, incidental findings

## Abstract

The identification of microbiological infection is usually a diagnostic investigation, a complex process that is firstly initiated by clinical suspicion. With the emergence of high-throughput sequencing (HTS) technologies, metagenomic analysis has unveiled the power to identify microbial DNA/RNA from a diverse range of clinical samples (1). Metagenomic analysis of whole human genomes at the clinical/research interface bypasses the steps of clinical scrutiny and targeted testing and has the potential to generate unexpected findings relating to infectious and sometimes transmissible disease. There is no doubt that microbial findings that may have a significant impact on a patient’s treatment and their close contacts should be reported to those with clinical responsibility for the sample-donating patient. There are no clear recommendations on how such findings that are incidental, or outside the original investigation, should be handled. Here we aim to provide an informed protocol for the management of incidental microbial findings as part of the 100,000 Genomes Project **which may have broader application in this emerging field. As with any other clinical information, we aim to prioritise the reporting of data that are most likely to be of benefit to the patient and their close contacts. We also set out to minimize risks, costs and potential anxiety associated with the reporting of results that are unlikely to be of clinical significance. Our recommendations aim to support the practice of microbial metagenomics by providing a simplified pathway that can be applied to reporting the identification of potential pathogens from metagenomic datasets. Given that the ambition for UK sequenced human genomes over the next 5 years has been set to reach 5 million and the field of metagenomics is rapidly evolving, the guidance will be regularly reviewed and will likely adapt over time as experience develops.

## Introduction

### Changes in sequencing technology

The emergence of high-throughput sequencing (HTS) over the last decade has revolutionised the area of genomic medicine. Since 2014-2015, the $1000 genome is a reality ^2^; as human genome sequencing becomes more affordable, it is likely to play an increasing role in routine clinical practice.

### Structure of the 100,000 Genomes Project

The Genomics England 100,000 Genomes Project reflects the increasing role of HTS and genomic medicine in routine clinical practice. It was designed to underpin development of clinical practice in the National Health Service (NHS) of the United Kingdom by fostering the genomics-based development of personalised and precision medicine. The project further strengthens the synergy between clinical investigation and research, aiming to accelerate participants’ benefit from HTS of their genomes. Recruitment was completed in 2018 and the de-identified (or depersonalised as all direct identifiers are removed from the dataset) sequence database is now available to groups of researchers forming the Genomics Clinical Interpretation Partnership (GeCIP) domains ^3^. On October 2018 an expansion of the 100,000 Genomes Project was announced to see 1 million genomes sequenced by NHS and UK biobank within the following 5 years, while the overall ambition for UK was set to 5 million genomes within the same 5-year period ^4^.

Participants (numbering approximately 85,000) in the 100,000 Genomes Project were NHS patients with a rare disease (plus in some cases family members) and patients with cancer. They were recruited by a healthcare professional involved in the patient’s clinical care and referred to 13 specialised NHS Genomic Medicine Centres. Genome sequencing was conducted to search for information that could potentially benefit the patient as well as contributing to research in that disease area. For the purposes of this paper, all participants will be referred to as patients, as the primary aim of the project is to advance clinical care.

### Consent and return of findings to participants

The term “health-related findings” is used in the current literature to describe a finding made by researchers during the course of a study that has potential health or reproductive importance to an individual participant ^5^. Amongst these findings can be “incidental findings” that are outside of the focus of the original investigation. The ethical issues raised by such findings have been considered extensively by other expert groups, whose opinions we have reviewed ^5– 11^. Participants in the 100,000 Genomes Project are already having primary findings directly related to their condition reported to them. At the time of recruitment, they could also choose whether to opt-in or not to receive “additional findings”, namely those for a limited set of conditions not connected directly to their primary diagnosis. Table 1 lists the conditions for which additional findings might be reported, although participants were also told this list might change as evidence about such findings accumulated.

**Table 1.** Genomics England additional findings list.

**Bowel cancer predisposition:**
*MLH1* (adult only), *MSH2* (adult only), *MSH6* (adult only), *MUTYH* (adult only)
*APC* (adult and child)
**Breast and ovarian cancer predisposition:**
*BRCA1* (adult only), *BRCA2* (adult only)
**Other cancer predisposition:**
*VHL* (adult and child), *MEN1* (adult and child), *RET* (adult and child)
**Familial hypercholesterolaemia:**
*LDLR* (adult and child), *APOB* (adult and child), *PCSK9* (adult and child)

This specified list of conditions was based on generally agreed requirements ^5^: these are recognized, serious conditions where a treatment approach exists that has been proven to be effective. In contrast, “incidental findings” were defined as findings that were generated without intent by the whole process rather than specifically sought. Simply because they have not been looked for does not mean they are not important; however, searching for every potential finding that might or might not be significant to the individual is time-consuming, expensive and may not produce beneficial information. Genomics England, in choosing its list of additional findings, has narrowed down the possible list of findings, consideration is now needed as to whether incidental microbial sequence findings constitute situations that should be communicated to participants and their clinical teams.

### Identification of microbial DNA as a secondary consequence of human genome sequencing

The 100,000 Genomes Project uses biological samples (whole blood or sputum) to determine the germline genome of each recruited patient (as well as tumour tissues for determining the cancer genome where applicable). The project takes a ‘re-sequencing approach’, whereby fragments of human DNA are read with HTS and subsequently mapped to an existing reference sequence of the human genome. Every piece of DNA present in the sample is equally likely to be read by HTS approaches. The indiscriminate reading of within-sample DNA suggests that nucleic acid unrelated to the human genome will also be included in the raw reads (unmapped to the human reference genome). Recognising that the presence of non-human DNA from microbes (bacteria, fungi, protozoa and viruses) could have clinical significance in specific circumstances, the Integrated Pathogen and Mobile Element GeCIP domain was formed in order to extract metagenomic microbial DNA from the 100,000 Genomes Project data. Whether the identification of microbial sequences in these data should be reported to the patient and the attending clinician to trigger a clinical investigation is a complex decision involving bioinformatic, technical, clinical and ethical considerations.

Early detection and treatment of harmful and potentially transmissible infections could help to improve health outcomes for participants and their close contacts. There is a balance between the need to have a robust reporting strategy in place for cases where participants and their clinical teams would benefit from receiving the information, versus avoidance of generating unnecessary investigations, anxiety and cost that could be generated by spurious, irrelevant or misleading results. Previous experience has suggested that medically relevant, actionable findings were found in between 0.8% and 5% of research participants ^12, 13^.

Exploring the nature and significance of microbial reads arising from metagenomic datasets is important. In response to the request of Genomics England Science Advisory Committee, we have drawn together colleagues with experience in medical microbiology and infectious disease, sequencing, bioinformatics and bioethics, to examine the issue of incidental microbial findings in human genomic DNA metagenomic data. Specifically, we have considered:

- The potential additional findings that may be generated by the Integrated Pathogen and Mobile Element GeCIP domain;
- The uncertainties associated with technical validity of pathogen detection within human samples;
- The practical criteria that might be applied to help define clinical ‘actionability’;
- The findings that should be communicated to the patient and to their clinical team, and in which circumstances this should occur.
- Findings which do not justify or need onward communication.

Through these discussions, we have advised on a process for the reporting of additional microbial findings. Our strategy seeks to strike a delicate balance – enabling patients to receive the best care based on available knowledge while not burdening them or their clinical teams with information that does not inform investigation, treatment or prognosis, and might result in unneeded additional interventions. Given that, as already stated, this project deliberately inhabits the hybrid territory between research and clinical practice, some of the difficulty in striking this balance arises from translating research findings (and a lack of large-scale evidence) into clinical practice.

## Considerations made by the expert committee

### The ability of metagenomic approaches to identify pathogens in clinical samples

***Technical assessment of the accuracy of metagenomic sequencing for diagnosis of infection.*** Diagnosis is usually based on a combination of clinical evaluation supported by laboratory testing and/or other investigations (e.g. imaging). A clinical investigation assesses the possibility that a patient indeed has or doesn’t have a specific disease. The pre-test odds for a patient having a disease are usually provided by epidemiological studies (e.g. cross-sectional studies of disease in a specific population) ^14^. For example, the risk of developing breast cancer within the age group 30-40 is 1 in 228 (data from https://www.breastcancer.org); thus a patient with no symptoms aged 35 has a pre-test odds for breast cancer of 1/227. These pre-test odds can be modified by the initial clinical assessment, which includes medical and family history and identification of signs and symptoms. Targeted laboratory testing then further modifies the pre-test odds. Once a positive test result has been acquired, a likelihood-ratio value of the positive test will upgrade the pre-test odds of having the disease as follows:

Post-testodds=pre-testodds×likelihoodratio

The likelihood ratio of a positive test is formally calculated by the ratio of the sensitivity of the test divided by (1-specificity)

Likelihoodratio=sensitivity/(1-specificity)(A)

Thus, in order to evaluate the ability of metagenomics findings to diagnose infectious disease in genomic data (including the 100,000 Genomes Project data) we would ideally evaluate the Likelihood ratio of the approach. According to Formula A an assessment of sensitivity and specificity of the approach compared to existing clinically validated approaches is required. It is anticipated that the approach will not have the same values of sensitivity and specificity for all pathogen findings, and output will be modified by other variables such as host immunity, sample type, and pathogen burden.

***Comparison of metagenomic data to existing clinically validated methods for pathogen identification at the species level.*** Here we consider the part of identifying a pathogen that is classifying it into a specific species. Sequencing followed by phylogenetic reconstruction is a powerful approach to classify new species as well as identify known species ^15, 16^. Alternative traditional approaches include: a) microbial cultures, b) nucleic acid tests (e.g. real-time PCR), c) identification of antigens and/or antibodies. The value and power of metagenomic data in identifying viruses has been recognized and proposed to be adapted from the International Committee of Taxonomy of Viruses (ICTV) ^17^. It is thus expected that under specific circumstances genomic data can provide a reliable way to identify a pathogen at the species level.

***Specificity and sensitivity of metagenomic data for identification of micro-organisms at the species level.*** Data generated as part of the 100,000 Genomes Project are comprised of “reads” (nucleotide sequences) of ∼150 bp in length ^3^. In theory, if the 150-bp-read shares high genetic similarity to a pathogen reference genome, this could represent highly specific identification of the pathogen. However, if the same read shared high similarity with another species, then the resolution at species level would be unreliable. A phylogenetic analysis of the candidate 150-bp-read sequence, together with reference sequences of closely related species on potentially homologous (or highly similar) genomic regions, could robustly indicate whether there is sufficient information to classify the sequence for a specific organism ^18^.

The sensitivity of the metagenomic approach (the probability of identifying the organism given its existence in the biological sample) is difficult to estimate. HTS is a theoretically unbiased approach, and we do not have any expectation that pathogen DNA molecules would have a different probability of being sequenced compared to human DNA molecules; however, this is based on the relative abundance of each sequence. Thus, in a mixture of pathogen/human DNA molecules in which human DNA accounts for the vast majority of sequence material, then the possibility of sequencing a pathogen sequence could be very low. This results in a low sensitivity of the approach for detecting the organism, as demonstrated by previous studies ^19^, but depends on the sample type and the burden of the organism. Some empirical examples are mentioned below:

a) The cerebrospinal fluid (CSF) sample of a person with pneumococcal meningitis could contain a high ratio of organism-to-human DNA ratio because the genomic DNA load of the CSF is lower than blood or tissue samples (low cell count which increases in bacterial meningitis), and the bacterial load of pneumococcal meningitis is usually high.
b) HIV infection with low pro-viral loads in blood stream would have a low ratio of organism-to-human reads, since whole blood has a high host genomic DNA load.
c) Adenovirus in respiratory secretions could be vastly outnumbered by commensal (bacterial) flora and human DNA.

Based on these considerations, it is feasible that metagenomic data have the potential to provide powerful identification of a pathogen at the species level and if performed appropriately the identification of the micro-organism could approach 100% specificity ^17^. In this case, identification is defined as the process of correctly assigning the species ID of an organism, which is only one component of the diagnostic process of identifying a pathogen that is clinically significant. The HTS approach as a diagnostic process may often suffer from low sensitivity, but due to the ultra-high specificity of the data it may still be valuable for diagnostic purposes: When specificity is 1, the likelihood ratio of the test becomes infinite regardless of the sensitivity of the approach (see Formula A), thus the certainty for the ID of the micro-organism is maximised.

The use of short sub-genomic sequences (e.g. 150 bp) in resolving pathogen species is expected to be more accurate in viruses than in bacteria and fungi. This is based on a number of features of viral genomes:

1) Viruses overall evolve fast ^20^, and phylogenetic information in homologous, non-repetitive regions can be sufficient to resolve evolutionary relationships even within the same species. In contrast, mutation rates (when considered per nucleotide) in bacteria and fungi vary a lot, with many important genes being under strong negative selection. Thus, short sequences (150 bp) can be insufficient in many cases for resolving evolution at the species level (e.g. ribosomal RNA ^21^).
2) Only large-genome (>30,000 bp) viruses harbour transposons (repetitive regions); small genome viruses (the majority of pathogenic viruses) have their genomes packed in a way that transposable elements would be a rare phenomenon. On the other hand, repetitive regions are present in both bacteria and fungi.
3) Non-homologous regions of viral genomes (e.g. short tandem repeats, mononucleotide stretches) have very low content of evolutionary information, thus they cannot be used for resolving species. Small viruses do not have long stretches of such regions, while they are commonly present in both bacteria and fungi.

Based on the above considerations, 150bp sequences derived from viruses of genomes <30,000 bp should frequently yield sufficient information to resolve identification at the species level. On the other hand, larger viruses (such as Herpesviruses), have low-complexity areas in the genome that could be confused even with the human genome (e.g. short-tandem repeats with telomeres). Even more difficult is the discrimination between bacterial or fungal species; identification of these organisms at the species level based on short reads can be difficult, unless the identified 150-bp read comes from specific genomic regions, or can be reconstructed with other sequence fragments to represent a longer continuous portion of pathogen genome.

**Specific recommendation**: The identification of pathogens within HTS can be highly specific, but may frequently be insensitive, and the clinical utility of these findings has not been validated, yet. Therefore, prospective analysis of pathogen sequences from metagenomic analyses is needed to determine likelihood ratios for diagnosis of infectious diseases. It is also noted that this might not be feasible (or clinically useful) for all available pathogens, thus we propose to prioritize pathogens according to their potential for clinical benefit. Considerations on the potential clinical benefit of pathogens should be examined by an Infectious Disease Expert subgroup. 

### Microbiological considerations beyond identification of pathogen at species level

The species identification of a pathogen is not sufficient to provide a clinical diagnosis. In order to upgrade the identification of a pathogen to a clinically important result we need to:

1) Evaluate sources of bias/uncertainty in the metagenomic process involving a pipeline from sample collection through the wet lab process and bioinformatic analysis (outlined in 3.2.1);
2) Evaluate the finding under a clinical microbiological framework (outlined below in *Evaluation under a clinical microbiology framework*);
3) Consider the result that has been obtained in light of the clinical sample, the patient condition and underlying diagnosis, and other relevant results (routine observations, imaging, laboratory parameters) (outlined below in *Evaluation under a clinical microbiology framework*).

***Sources of uncertainty in the generation and interpretation of metagenomic data.*** Pathogen metagenomic findings should be examined as a laboratory procedure, thus we need to consider potential sources of error.

(i) Pre-analytical errors: Genomics England samples have been collected according to a validated procedure for human genome sequencing, including aseptic technique which should result in minimal pre-analytical errors for pathogen isolation. However, the approach has been validated for collection of human DNA, not for microbial diagnostics. Thus, there is still potential for contamination of samples by micro-organisms (most likely bacteria or fungi). Organisms that are commonly found in the environment or as part of human flora are more likely to be found due to pre-analytical contamination, but contamination of reagents used throughout the wet-lab process has also been described ^22, 23^ (see also *Extended data*, extended text ^24^).

(ii) Analytical errors: these errors are introduced either as a result of the sequencing process or based on bioinformatic analysis. Due to the high fidelity of Illumina sequencing, the probability of the former is low. As discussed in below in *Relevance of pathogen findings with patient’s recruitment and well-being*, metagenomic data can result in specific identification of a pathogen but can also lead to indeterminate conclusions about the presence of a specific micro-organism (also discussed in *Extended data*, extended text ^24^ and below in *Relevance of pathogen findings with patient’s recruitment and well-being*). We thus suggest the following criteria to be taken into account when considering the identification of a pathogen based on metagenomic data:

a) Sequence specificity: In order to confirm that the sequence of a microbe is present in the unmapped HTS data (i.e. data that cannot be aligned with the human genome), the query sequence should be uniquely mapped to the microbial genome. This can be investigated through a standard search (e.g. BLAST ^25^) of the query sequence against a full database of existing microbial sequences, which should retrieve the specific microbe as the top hit with a significant divergence from the second hit. This should be followed up by phylogenetic analysis to confirm clustering of the query sequence within the identified species, supported by high bootstrap values (>75) (phylogenetics are also discussed above in *The ability of metagenomic approaches to identify pathogens in clinical samples*).

b) Number of sequences: Many microbes are commonly found in the environment or as part of normal human flora. Even with robust aseptic technique, the procedure of obtaining a blood or tissue sample is potentially subject to contamination. Such contamination should usually be low-level, and thus quantitative support (e.g. number of DNA sequences) may help to differentiate between low-level contamination vs. the presence of a genuine infecting agent. Considerations about environmental contamination of the received sample in the post-sampling pre-analytical period have been reported ^26^ and should also be taken into account ^27^. Cross-contamination during the different stages of laboratory processing, from extraction to sequencing, can result in ascribing a result to the wrong sample. Cross-contamination can be intra-run or inter-run thus knowledge of pathogens normally processed by the lab can be useful. One way to test for potential contamination is by performing phylogenetic analysis of retrieved pathogen sequence data in the same run and immediate previous runs: unexpected closely related pathogen sequences between unrelated participants would be evidence of post-sampling contamination ^28^. With respect to defining a threshold of number of reads above which the identification of the microbe in the sample is considered very likely, we comment below in *Evaluation under a clinical microbiology framework*.

***Evaluation under a clinical microbiology framework.*** Results should be evaluated based on the source of the biological sample, recognising the presence of commensal and/or environmental flora in some sample types (e.g. stool, sputum) versus the anticipated sterility of others (e.g. blood, CSF, joint aspirate). Thus, it is important, especially for non-sterile sites, to consider the need for quantitative thresholds above which we may consider that the presence of specific microbial sequences is more likely to suggest the presence of clinical infection.

The presence of microbes that are known to be part of normal flora should be interpreted cautiously; the combination of background information (e.g. biological sample, clinical context) would be required to determine clinical significance. For example, microbial cultures sometimes require the quantification of the microbe to help distinguish between contamination or low-level (non-pathogenic) microbial colonization (e.g. sputum or urine cultures, in which a typical threshold of >10 ^5^ cfu/ml is typically assigned for clinical significance). With respect to distinguishing colonization from an active infection, some microbial cultures require quantification of other features of the sample, such as number of white blood cells. The latter can help to distinguish between microbial infection and benign colonization. Such clinical sample data are missing from Genomics England samples, thus clinical interpretation for non-sterile samples needs to be examined very cautiously.

Data generated as part of the 100,000 Genomes Project are strictly genomic DNA. Thus, RNA molecules with no DNA stage (such as RNA viruses, not including retroviruses) should not currently be reported as they are most likely to represent artefacts. However, in selected cases where there have been reports of reverse transcription of microbial RNA from active human transposons, such as for example in HCV ^29^, the identification could be reported to the clinician/patient as it might signify the existence of an integrated virus in the context of a true infection.

***Timing of data availability.*** The timing of data analysis generated by the 100,000 Genomes Project typically occurs weeks to months after the sampling process. On this basis, we determined that all microbes identified and known to be responsible for acute-syndrome pathogenesis without chronic sequelae should not be reported to the patient or clinician, as their identification would not contribute to the clinical management of the patient. For example, bacteraemia is usually an acute short-term illness; the identification of bacteria in blood samples that have been drawn 6 months before the analysis is rarely likely to provide any clinical benefit to the patient. In contrast, microbes associated with chronic syndromes could provide a benefit if reported to the clinician and the patient, as they might remain undiagnosed and continue to contribute to pathology.

Finally, microbes of public health importance could potentially trigger an epidemiological investigation. The timing of the identification is crucial in cases of public health importance; thus, it should be considered whether an epidemiological investigation several months after initial identification is likely to have a benefit, and to weight this against the resources required for an investigation, and potential unnecessary anxiety to the public patient.

***Prevalence of a microorganism in human populations.*** Under the presupposition that participants’ symptoms were not considered relevant to infectious diseases, identification of pathogens that are highly prevalent in the general population provide a small margin of diagnostic benefit. The pre-test odds of having the infection is high (i.e. even before our metagenomic analysis), thus an increase of these odds as a result of the metagenomic findings is unlikely to enable a diagnostic benefit. For example, a pathogen such as Epstein–Barr virus has a prevalence of 90% in some populations. A test would update the *a priori* probability of infection from 0.90 to close to 1 (i.e. 10% increase of the probability of having the infection). Given the uncertainties in the metagenomic approach (i.e. follow-up clinical test verification is required), the probabilistic gain in diagnosis is very low. If the finding is actually important for patient’s well-being, then high pre-test probabilities should have necessitated clinical investigation especially through general population screening recommendations.

***Pathogenicity of a microorganism.*** Reporting low-pathogenicity organisms (e.g. pathogens which are unlikely to impact the patient in the long-term) is less likely to enable a benefit, although this may depend on the clinical status of the individual (see also *Clinical context of the research participant*, below). Pathogenicity varies according to host context, and the type of sample from which the organism is identified. However, some organisms (e.g. HIV) are considered pathogenic irrespective of the sample type or clinical details of the host, and their identification should trigger a clinical investigation regardless of the patient’s clinical status. On these grounds, reporting highly pathogenic organisms is likely to be beneficial.

***Potential for treatment and/or prevention.*** In the absence of guidelines for treatment/prevention of specific infections, reporting them is unlikely to enable a clinical benefit (e.g. for most viral infections within the community). However, if knowledge of the presence of the microbe is important for the prognosis within the individual host, or could have a benefit to sexual partners, other close contacts and the unborn child, this could be reported even in the absence of potential antimicrobial intervention.

***Clinical context of the research participant.*** Co-morbidities, including immunosuppression, may be a relevant consideration. Many infections in immunocompetent individuals (including most viral infections) are less likely to have long-term clinical significance or underpin any treatment decisions.

**Specific Recommendation**: We propose the formation of an Infectious Disease Subgroup comprised of clinical microbiologists and infectious disease specialists. The purpose of this subgroup was to evaluate potentially important microbial findings through the process adopted by Genomics England for reporting additional findings before communicating to the clinician. The Infectious Disease Subgroup reviewed an extensive list of potential human pathogens ( *Extended data*, Pathogen list ^24^) and proposed which metagenomics pathogen findings should be followed up in this way (see below, *Specialist considerations made by the Infectious Disease Subgroup*).

### Relevance of pathogen findings with patient’s recruitment and well-being

***Reporting primary and additional findings.*** At present, there are insufficient data to determine the extent to which certain microbes could be directly related to the patient’s index condition or those under the additional findings list. While exposure to microbes is a major cause of cancers – epidemiological data suggested that in 2002 17.8% of cancers were caused by viral (∼12%), bacterial (5.6%) and helminth (0.1%) infections ^30^ – establishing primary causation may be much more difficult. The International Agency of Research for Cancer ^30^ has categorized biological agents into four categories according to evidence supporting their role in causing cancer ( Table 2).

**Table 2.** Carcinogenicity of biological agents ^30^.

Group	Carcinogenicity
1	The agent is carcinogenic to humans
2A	The agent is probably carcinogenic to humans
2B	The agent is possibly carcinogenic to humans
3	The agent is not classifiable as to its carcinogenicity to humans
4	The agent is probably not carcinogenic to humans

The subgroup considered whether category 1 microbes should be reported to cancer patients as they could potentially be considered as “primary findings”. According to information provided to patients during the consent, the majority of participants should have understood that a “primary finding” would be a feature of the human genome, and may not expect their data to include microbial sequences. Crucially, many such agents are ubiquitous in most human populations, and evidence of exposure to a micro-organism would not inform management of an established cancer (as exemplified by herpesviruses and human papillomavirus).

**Specific recommendation**: At present, there is insufficient evidence that oncogenicity of a microbe, apart from hepatitis B virus (HBV) and hepatitis C virus (HCV), should mandate reporting of the identification of that microbe to cancer patients.

***Reporting incidental microbial findings.*** As discussed, incidental microbial findings may have significant importance for an individual. This has been the case for non-microbial (genomic) incidental findings, for example mutations associated with cystic fibrosis or Huntingdon’s disease in a cancer patient are not related to the reason that the patient was recruited, but can be important for their own well-being and/or that of their family and future offspring. If an incidental finding is discovered (microbial or not-microbial) decisions will need to be made whether it is important to the health of the individual and whether and how this should be communicated.

**Specific recommendation**: We recommend evaluating potentially important incidentally-discovered pathogen metagenomics findings for their impact on the health and well-being of participants and their families (i.e. a personalized assessment of the finding given information available for the participant).

### Bioethical considerations and consent

When a proportion of participants agreed to receive “additional findings” that are not relevant to their primary condition, it is unlikely that they considered that this might include the identification of infectious agents that are not directly related to their index diagnosis. For future studies in which metagenomic data are collected, consent discussions should provide a range of potential additional findings (including detection of pathogens) and emphasise that communication will usually be decided by a multidisciplinary team, based on both evidence and risk/benefit assessment.

Some participants chose not to receive any additional findings. However, experience has shown that participants often couldn’t accurately recall what decision they had made, but they expected clinically useful information to be reported back. One approach to maintaining up-to-date valid consent could be to re-contact participants at regular intervals to check their wishes; another option would be to ensure that future consent was broad enough to cover this sort of possibility. Providing patients with general data about study results may prompt individuals to re-examine their choice. Evidence from Genomic Medicine Centres suggests that many people do not recall what decision they made regarding additional findings, nor that they understood what might be revealed in this category (personal communication).

**Specific recommendation**: Although there is insufficient evidence that opting out from feedback of additional findings constitutes a valid advance refusal for infectious disease incidental findings, if the findings may have a health-related benefit, they should be passed to a clinician for further examination.

### Conclusion of Expert Committee

Given the high volume of data generated as part of the 100,000 Genomes Project, clinical microbiology follow-up of every potential pathogen finding is not feasible. We thus propose filtering out potentially spurious or low priority, clinically unimportant results of the metagenomics approach, in order to allow the infectious disease subgroup to focus on high-priority findings (as recommended in *Suggested pathogens to be reported for further clinical evaluation*).

## Specialist considerations made by the Infectious Disease Subgroup

### Suggested pathogens to be reported for further clinical evaluation

The infectious disease subgroup, after considering the domains outlined in the preceding sections, unified by considering the overall importance of the findings for the participant’s welfare and the likelihood of the results being significant to clinical management, reviewed an exhaustive list of potential human pathogens ( *Extended data*, Pathogen list ^24^).

They concluded that, for the time being, the following list of pathogens should be flagged as potentially important for the participant’s welfare and that of their close contacts:

1) HIV
2) HBV
3) HCV
4) Human T-Lymphotropic Virus

A summary of the considerations is provided in Table 3. Infections of these viruses comply with our fundamentally agreed criteria of being persistent, serious, treatable (actionable) and/or transmissible. They are also unlikely to be the result of environmental contamination (these pathogens replicate within human cells) thus the pre-analytical source of bias is minimized. Finally, their genomes are sufficiently complex and distant from other non-pathogenic microbes so that their metagenomic identification is likely to be reliable.

**Table 3.** Summary of considerations for reporting metagenomic findings.

Consideration	Point of Relevance	Criteria for reporting to research participant	Recommendation
1. Need to discriminate between environmental contamination, and true presence of a microbe in a clinical sample;	Pre-analytical	There must be reasonable certainty that an organism identified is a pathogen	Organisms that may reflect environmental contamination should not be reported.
2. Need to discriminate between commensal flora (harmless to the host) vs pathogenic organisms (associated with a disease phenotype);	Pre-analytical Clinical evaluation	There must be reasonable certainty that an organism identified is a pathogen	Organisms that may represent commensal flora should not be reported. Reporting should be reserved for organisms that cause serious and persistent infection.
3. Long time delays may occur between acquisition of a clinical sample and identification of microbial reads through the metagenomic pipeline;	Clinical evaluation	Identification of a pathogen must occur within a period deemed timely for appropriate clinical intervention	After a time lapse of weeks/months, feedback to the participant is not relevant for the majority of pathogens. Reporting should be reserved for conditions that influence treatment decisions.
4. Quality controls are required for metagenomic sequencing of pathogen DNA;	Analytical	Sequencing platform is currently not validated for clinical diagnostics, so the majority of pathogen sequence data should not be reported to participant	Over time, consider developing positive controls for microbial identification from metagenomic sequencing platforms. Sterility of sample handling and sequencing environment needs to be assured.
5. Quality controls required for bioinformatic processing of sequencing data;	Analytical	Bioinformatic platform is currently not validated for clinical diagnostics, so the majority of pathogen sequence data should not be reported to participant	Over time, pathogen-specific and tissue- specific algorithms are required (e.g. to specify what number and % of reads, length and depth of coverage of a micro-organism’s genome) to report its presence in the sample. A designated pathogen database of reference genomes should be developed
6. Identification of a pathogen may have implications for contacts of the participant;	Clinical Evaluation	Confirmation of a specific pathogen would have consequences for testing/ treating family members or sexual contacts	Identification of blood-borne viruses should be reported, as contacts/family members should be offered screening ± treatment if infection is confirmed
7. Special considerations may arise if biohazard organisms are identified or reported (potential impact on laboratory staff, patient’s clinical teams, family members, and the wider community);	Clinical Evaluation	There must be reasonable certainty that an organism identified is a pathogen, has been correctly identified, within an appropriate time-frame for clinical action	Reporting biohazard organisms could cause high anxiety and cost affecting a wide number of people. Reporting should only be undertaken in keeping with points 1–6 above.
8. Need to differentiate between results that may have impact for an individual patient, versus those that could underpin future patient-stratified medicine or provide longer-term insights into pathophysiology of disease	Clinical Evaluation	The presence of a micro- organism should only be reported to the participant/ clinician if it will change current clinical management.	Organisms that may be important in disease pathogenesis (e.g. oncoviruses) should be recorded for research purposes but not reported back to individual participants.

###  Clinical evaluation, audit and communication with patient

**Figure 1.**
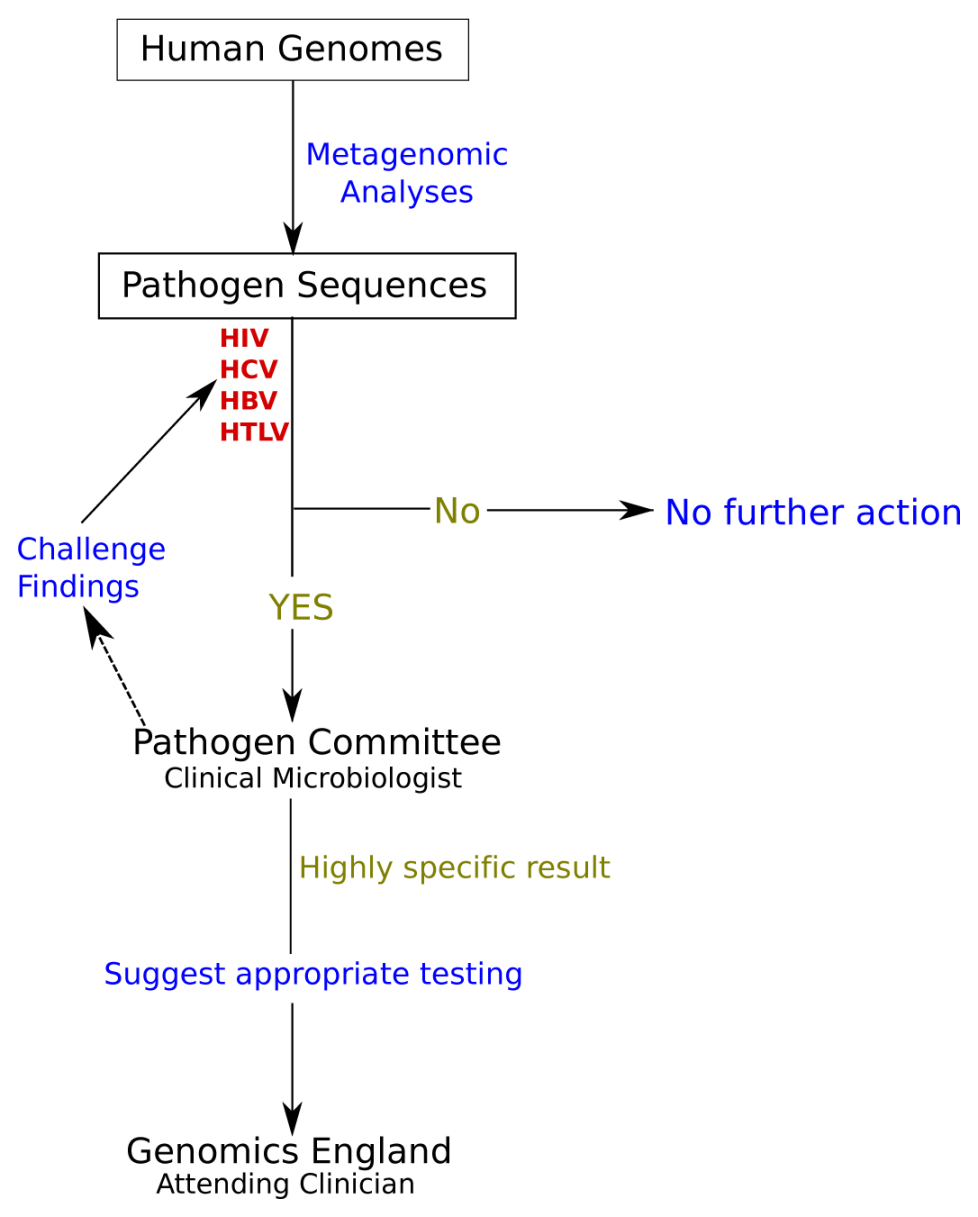
Suggested pathway for reporting potentially important pathogen findings from the 100,000 genomes project projected and potentially adapted for other human whole genome sequencing projects. Dotted line shows an optional pathway. Blue=actions taken, Green=results obtained, Red=queries made.

When the identification of the pathogen is considered highly likely, then ideally it should be evaluated by a clinical microbiologist who would put this finding into appropriate context before discussion with the patient’s attending clinician ( Figure 1). Until metagenomics findings have been properly validated producing outputs sufficiently simplified for clinical microbiologists, the role of the clinical microbiologist will be covered by the Infectious Disease Expert Subgroup. Given the position of clinical microbiologists in NHS infectious disease services, we propose the following process of microbiological investigation:

a) evaluation of pathogen findings and if appropriate request additional validation (such as independent bioinformatic analysis to be performed on the data),
b) evaluation of the importance of the findings for the patient’s welfare in line with considerations detailed in this report,
c) suggestions with respect to appropriate routine testing for validation of the result

Since data are depersonalized for researchers and the Infectious Disease Subgroup, but can be linked with specific patients through Genomics England, the result, if deemed sufficiently valid, will then be communicated with specific recommendations for follow-up to Genomics England. Genomics England has designed specific procedures for reporting additional findings to the clinician who will then have the duty of care to schedule appropriate follow-up with the patient and planning further clinical investigation as required.

### Suggested future actions

The list of the pathogens will be reviewed by the 100,000 Genomes Project Infectious Disease Subgroup based on research findings from the Integrated Pathogen and Mobile Element GeCIP, as well as evidence released by other groups as more data become available. The policy and microbial data generated so far and, in the future, will be kept under regular review (suggested as every 6-12 months) by the Infectious Disease Subgroup.

## Conclusions

High-throughput sequencing has created opportunities to advance healthcare. The new genomic medicine service for the NHS is founded in the 100,000 Genomes Project. Genomic data generated through this process have the potential to be valuable in ways that we do not yet fully understand and had not fully anticipated at the outset. Here, we have considered the potential for metagenomics analysis to generate pathogen-specific health related findings. We have aimed to strike a balance based on current evidence, clinical relevance to participants and their families, and burden of uncertain findings ( Table 4). Sequencing technology has advanced more rapidly than our ability to interpret much of the data that are generated. We need to be prepared to reassess and amend our recommendations in the light of new evidence or understanding, ensuring patient and public involvement in our process, and avoiding hype or tick box approaches to disclosure.

**Table 4.** Overall recommendations.

Recommendation number	Recommendation
1	Pathogen sequences from metagenomic analyses should be analysed prospectively to determine likelihood ratios for diagnosis of infectious diseases.
2	An Infectious Disease Expert subgroup, which should be comprised of clinical microbiologists and infectious disease specialists, should be responsible for reviewing the potential clinical benefits of reporting pathogens to clinicians
3	Based on existing insufficient evidence, oncogenics microbes, apart from HBV and HCV, should not be reported to cancer patients.
4	If findings may have a health-related benefit, they should be passed to a clinician for further examination and possible communication to patients
5	*Incidentally* discovered pathogen metagenomics findings should be evaluated from Infectious Disease Expert subgroup for their potential impact on the health and well-being of patients and their families and whether they should be reported.
6	The below pathogens should be flagged as potentially important for the participant’s welfare and that of their close contacts:
a	HIV (Human Immunodeficiency Virus)
b	HBV (Hepatitis B Virus)
c	HCV (Hepatitis C Virus)
d	HTLV (Human T-Lymphotropic Virus)
7	This policy and the microbial data generated should be kept under regular review (suggested as every 6–12 months) by the Infectious Disease Subgroup based on research findings from the Integrated Pathogen and Mobile Element GeCIP as well as emerging evidence from scientific literature.

## Data availability

### Underlying data

No underlying data are associated with this article.

### Extended data

Harvard Dataverse: Extended Text for "Potential for diagnosis of infectious disease from the 100,000 Genomes Project Metagenomic Dataset: Recommendations for reporting results". https://doi.org/10.7910/DVN/ADJGKX ^24^.

This project contains the following extended data:

- Extended text.
- Pathogen list (list of pathogens considered for reporting).

Extended data are available under the terms of the Creative Commons Zero "No rights reserved" data waiver (CC0 1.0 Public domain dedication).
