# Supplementary material for: Potential for diagnosis of infectious disease from the 100,000 Genomes Project Metagenomic Dataset: Recommendations for reporting results
Source: Wellcome Open Res. 2019 Oct 14;4:155. [Version 1] doi: 10.12688/wellcomeopenres.15499.1 (PMC6993825; doi:10.12688/wellcomeopenres.15499.1)
Supplement: Peer review history

# Peer review: Reviewer response for version 1

**DOI:** 10.21956/wellcomeopenres.16959.r37488

**Published:** 2020-02-27

**Authors:** Alejandro Sanchez-Flores

**Affiliations:** 1 Unidad Universitaria de Secuenciación Masiva y Bioinformática, Instituto de Biotecnología, Universidad Nacional Autónoma de México (UNAM), Cuernavaca, Mexico

It would be important to have a specific reference (REF 5) for definitions like "findings" terms. Even if some of these are explained, a glossary or a reference where all these findings could be found would help as a guide for both participants (or patients) and researchers (or MDs).

 Page 3, in the phrase:

 "In contrast, “incidental findings” were defined as findings that were generated without intent by the whole process rather than specifically sought. Simply because they have not been looked for does not mean they are not important; however, searching for every potential finding that might or might not be significant to the individual is time-consuming, expensive and may not produce beneficial information."

 I found it a bit contradictive. I would suggest removing that "may not produce beneficial information" since until it is explored, it might or might not be informative or useful. The idea of a massive study is that potential findings can be informative, but I agree that it is time-consuming and expensive to look for them.

 Table 1 - I guess that the term predisposition is the correct medical term, but I would suggest a bit more of explanation to avoid misconception by the readers. I agree with it in terms of defining and additional findings. Would the word "risk" be more suitable?

 There is a lot of referred (medical) information and terms that are cryptic, but currently is the only information available and is not the authors fault. For example, references 5 and 30, also refer to other (non-scientific) documents. In that sense, I think that the authors can elaborate more on the section "Suggested future actions", about how research and their findings will feedback the health system and how it will influence on policy makers in order to establish a virtuous cycle where personal genomics will change medicine and treatments in the near future.

I confirm that I have read this submission and believe that I have an appropriate level of expertise to confirm that it is of an acceptable scientific standard.

# Peer review: Reviewer response for version 1

**DOI:** 10.21956/wellcomeopenres.16959.r37405

**Published:** 2020-02-05

**Authors:** Meredith L. Carpenter

**Affiliations:** 1 Arc Bio, LLC, Scotts Valley, CA, USA

In this report, Magiorkinis and colleagues describe their group’s recommendations for reporting incidental findings of pathogen sequences in whole genome sequencing datasets, in particular from the UK 100,000 Genomes Project. Discussion of this issue is increasingly important as whole genome sequencing becomes more common in the clinic – while there are increasing numbers of metagenomics assays being developed specifically to detect pathogen sequences in clinical samples, how/whether to interpret sequences found in the background of existing whole genome sequencing datasets has not been as thoroughly explored.

 Overall, the authors provide a comprehensive overview of the challenges inherent in attempting to identify pathogens from sequencing data derived from blood, particularly with a sample preparation workflow that was not intended for or validated for pathogen identification. Without many of the controls and validations required to derive confidence in interpreting the data, any conclusions must be drawn with an abundance of caution. The resulting recommendations (i.e., to only report viruses that are relatively unique on the sequence level and that have long-term health consequences, and to follow up with orthogonal verification of any preliminary positives) are conservative and logical given these complexities.

 Specific comments:

- Page 4, second paragraph: There has been at least one previous major study that investigated the prevalence of background pathogen sequences present in datasets originally collected for whole genome sequencing (Moustafa *et al.*, 2017 ^1^); this should be discussed given the relevance of the data to these recommendations (e.g., what are the most common pathogens that are actually observed in these types of datasets, and at what levels?)
- The methods and databases used to identify pathogens in metagenomics datasets have a critical influence on the interpretation of the data. The authors provide several recommendations for bioinformatics analysis approaches and cutoffs (e.g. page 4, page 6), without much support or context for these recommendations. For increased utility by those analyzing similar data, the authors could have included more discussion of the bioinformatics platforms available for these types of analyses and potential guidelines for selecting a bioinformatics workflow for this purpose. Naïve BLASTing of non-human reads is not necessarily going to be the most accurate or appropriate approach here, and there are many options (both commercial and freely available) for a more robust analysis.
- Ultimately, any potential findings derived from a WGS dataset must be confirmed by a validated orthogonal method. This is addressed in the text, but this point could be emphasized in the context of deciding which results to follow up on.
- Page 3 (minor) – this sentence should be revised to replace the 3 ^rd^ comma with a semicolon or period. “Genomics England, in choosing its list of additional findings, has narrowed down the possible list of findings, consideration is now needed as to whether incidental microbial sequence findings constitute situations that should be communicated to participants and their clinical teams".

I confirm that I have read this submission and believe that I have an appropriate level of expertise to confirm that it is of an acceptable scientific standard.

## References

[1] : The blood DNA virome in 8,000 humans. *PLoS Pathog*.13(3) : 10.1371/journal.ppat.1006292 e1006292 10.1371/journal.ppat.1006292 28328962PMC5378407

# Peer review: Reviewer response for version 1

**DOI:** 10.21956/wellcomeopenres.16959.r37404

**Published:** 2020-01-28

**Authors:** Matt S. Zinter

**Affiliations:** 1 Division of Critical Care Medicine, Department of Pediatrics, University of California, San Francisco (UCSF), San Francisco, CA, USA

In this article, Magiorkinis *et al. *discuss a proposed framework for reporting the identification of microbial pathogens unintentionally detected in human metagenomic sequencing samples to patients from whom those samples were obtained. The authors appropriately review the potential benefits of disclosing detected pathogens to participants, which include the potential for pathogen-targeted therapy that may benefit the participant or future offspring, as well as the potential to limit disease transmission within a community. The authors also review the numerous potential risks of disclosing detected pathogens to participants, including risk for anxiety, fear, unnecessary medical testing, and cost. These risks are a by-product of the current inability to strongly associate the mere presence of a pathogen with the clinical state of infection in most participants. Despite potential pathogenicity, most microbes that will be detected by the 100,000 Genomes Project will be unable to be labeled as medically relevant pathogens given the time-delay between sample acquisition and results, the lack of detailed clinical information, and an as-of-yet unclear delineation between commensal and pathogenic states. In an effort to balance the potential risks and benefits of disclosing inadvertently identified microbes, the authors agree that four viruses (HIV, HBV, HCV, and HTLV) can be identified with high certainty and high chance for potential benefit to patients and society and thus should be disclosed. The process for disclosure will involve a separate panel of expert physicians and microbiologists to assure the validity of reported findings prior to disclosure. The authors propose that the potential disclosure of additional microbes will be reviewed on a routine basis as data from this (and other) cohorts mature.

 In summary, this article proposes a useful framework for deciding how and why to disclose certain microbiological findings from genomic sequencing samples to patients who donated those samples to research.

I confirm that I have read this submission and believe that I have an appropriate level of expertise to confirm that it is of an acceptable scientific standard.

# Peer review: Reviewer response for version 1

**DOI:** 10.21956/wellcomeopenres.16959.r37492

**Published:** 2020-01-21

**Authors:** Prasit Palittapongarnpim, Vorasuk Shotelersuk

**Affiliations:** 1 Center of Microbial Genomics, Department of Microbiology, Faculty of Science, Mahidol University, Bangkok, Thailand; 2 Thailand National Science and Technology Development Agency, Khlong Nueng, Thailand; 3 Department of Paediatrics, Faculty of Medicine, Chulalongkorn University, Bangkok, Thailand

**General Comments**

 This is a thoroughly thought through paper, and is timely written as more and more human genomes are becoming available from various sequencing projects from around the world. Appropriate action plans for reporting incidental discoveries of infectious diseases from human genome analysis are still lacking and thus are required. This work proposes a protocol how to prioritize and report microbial findings back to responsible medical personnel when incidentally discovered in human genome analysis. This smartly broadens the applications of whole human genome sequencing projects. The proposed recommendation would facilitate early detection and treatment of harmful and potentially transmissible infections. The authors have considered all significant and relevant aspects of this application. The presentation is informative, clear and step-wise, which helps readers understand various aspects of the issues well. Some useful recommendations are presented. Some discussions can be improved however, in particular about the challenges in interpretation of the pathogen detection results.

 The following comments should be considered as minor, more as possible additional points for future considerations and studies.

**Specific points**

1. Incidental discovery of infectious diseases is an important issue in human genome analysis, and the recommendations in this work can be applied to any human genome projects. Given its potentials, one could see that these recommendations may be adopted elsewhere outside the UK, especially since sequencing human genomes is becoming more commonly practiced as a routine medical procedure in many more countries. Nevertheless, the authors could briefly note to the readers that there might be several issues regarding adapting these recommendations to other human populations. Microbial genomes and distributions, even of the same species, are expected to differ for human populations that are in different geographical parts of the world. This modifies the prior probability, for example. Clinical implications of the discovery of particular microbes also vary across different human populations (with different genomic backgrounds). Applications of this idea to other countries, therefore, need their own bioinformatics, technical, and clinical validations. Pathogens suggested to be reported for further clinical evaluation are possibly of different sets for different countries also. In addition, laws and social norms are different in various countries; ELSIs are thus needed to be considered before adopting in another context.
2. **Page 5** **“** ***Based on the above considerations, 150bp sequences derived from viruses of genomes <30,000 bp should frequently yield sufficient information to resolve identification at the species level*** **”.** The authors do not cite any papers for this claim and personally, we find this statement rather unconvincing. There are many known cases of horizontal gene transfers among viruses of different, but closely related, subtypes, and thus using just 150bp sequences for pathogen identification can misidentify their species. As the authors mentioned in their work, ICTV recognizes that metagenomic analyses can be used for virus identification and even acknowledges that “ *viruses that are known only from metagenomic data can, and should be, incorporated into* *[* *their* *]* *official classification scheme*”, but ICTV requires that at least the whole genome is known. The authors should justify better why 150bp sequences are sufficient and why the whole genome is not needed for this task. In addition, the authors should suggest which genomic regions, if not the whole genome, should be used for pathogen identification. Given a small list of the focus pathogens (page 8), this should be possible.
3. **Page 6 and Table 3; considerations 1 and 2**. The authors raised a very interesting point that human tissue samples can be contaminated with “ *[* *o* *]* *rganisms that are commonly found in the environment or as part of human flora*”. The discovery of such organisms in human DNA samples, as the authors noted, might not be clinically relevant, and perhaps might be not be necessarily beneficial to communicate such findings to the patients. The authors discussed that some quantification data, for example, microbial culture and white blood cell concentrations, are useful for distinguishing between benign colonization and active infection, but they also acknowledged that such data is missing from the Genomics England. Although the cautiousness is appropriate, we do believe that, with some limitations, there are still things that bioinformaticians can do with metagenomic data to preliminarily distinguish microbial active pathogens from normal flora. Such protocol, which certainly would be useful and relevant, particularly in the absence of the other information.
4. **Page 6** **“ *[*** ***Pathogen sequence specificity*** ***]*** ***can be investigated through a standard search*** ***(*** ***e*** ***.*** ***g*** ***.*** ***BLAST ^25^*** ***)*** ***of the query sequence against a full database of existing microbial sequences, which should retrieve the specific microbe as the top hit with a significant divergence from the second hit*** ***.*** **”** We feel that this guideline is too naïve and is not quite precise enough which can lead to misunderstanding. For example, it is very common that there are multiple sequences of the same organisms in one database (even for the curated one like the non-redundant nucleotide collection NCBI database); thus, the best true positive hit might not be that different from the second one. Furthermore, e-value and percentage identity cutoffs, which are extremely important for distinguishing between authentic from false positive BLAST hits, are missing from the discussion.
  On the other hand, it is also possible that sequences of the pathogens in the tissue samples might not be present in the (NCBI) database, and thus, according to this criterion, might lead to false negative results. The authors should better discuss this aspect of pathogen detection methods and caveats.
5. **Page 6** **“ *[Similarity detection*** ***] should be followed up by phylogenetic analysis to confirm clustering of the query sequence within the identified species, supported by high bootstrap values*** ***(>75*** ***)*** **”**. This is very specific and sounds like a determinative recommendation for assessing sequence specificity. Please provide references to support that a clade support value of 75% is sufficient for species identification.
**Page 6** **“ *[*** ***C*** ***]*** ***ontamination should usually be low*** ***-*** ***level, and thus*** ***quantitative support*** ***(*** ***e*** ***.*** ***g*** ***.*** ***number of DNA sequences*** ***)*** ***may help to differentiate between low*** ***-*** ***level contamination vs*** ***.*** ***the presence of a genuine infecting agent*** ***.*** **” **We would like to note that some rare opportunistic pathogens, are encountered infrequently, possibly with rather low microbial loads in human genomic samples, and, therefore, under this criterion, can be easily falsely dismissed as contaminants. Its finding may be clinically relevant however. The authors should discuss this caveat, perhaps on Page 7, together with the discussions on the problems of opportunistic infections in immunocompromised hosts.
6. **Page 7 Paragraph 2,** **“** ***Finally, microbes of public health importance could potentially trigger an epidemiological investigation*** ***…*** **”**. Potential benefits of human genome analysis to public health disease control can be considered more here. For example, if low level of unusual but the same microbes are repeatedly found in the same settings, would that signify early spill-over infection from an unidentified common source or non-human sources (such as livestock) to human? When human genomic sequencing becomes more routine, will identifying early stage of animal pathogen adaptation to human be possible? To address these questions, there need to be ways to effectively compare the information across multiple samples, and this can be discussed here.
7. As suggested by the authors, it would be interesting to see if long-read sequencing technologies using to sequence human whole genomes (10,000s or million bp/read), can be used for identification of microbial agents at the species level.

We confirm that we have read this submission and believe that we have an appropriate level of expertise to confirm that it is of an acceptable scientific standard.
